# Hepatotoxicity After CDK 4/6 Inhibitor Initiation in the Treatment of Hormone-Positive Metastatic Breast Cancer

**DOI:** 10.7759/cureus.40871

**Published:** 2023-06-23

**Authors:** Kashmira Wani, Kunj Patel, Vrushali Dabak

**Affiliations:** 1 Internal Medicine, Henry Ford Health System, Detroit, USA

**Keywords:** hormone receptor-positive breast cancer treatment, hormone receptor-positive breast cancer, hepatotoxicity, cdk 4/6 inhibitors, metastatic breast cancer

## Abstract

Cancer cells proliferate using various mechanisms. One mechanism of preventing tumor cell growth is blockade of the cyclin-dependent kinase (CDK) 4/6 axis. Multiple CDK 4/6 inhibitors - ribociclib, palbociclib, and abemaciclib - have significantly improved progression-free survival rates. However, they can cause hepatotoxicity. We present a case of a 67-year-old female who was diagnosed with stage 1C invasive ductal carcinoma. She was treated with letrozole and ribociclib due to recurrence as metastatic disease, but within 10 days, she developed transaminitis. She then started palbociclib but experienced elevated transaminases within two weeks, needing discontinuation of palbociclib. Subsequent positron-emission tomography/computed tomography imaging showed disease progression, and she was started on fulvestrant. We considered adding abemaciclib, but the patient declined and has had stable disease for more than a year on fulvestrant. CDK 4/6 inhibitors are used to treat metastatic breast cancer and are generally well tolerated. The most common side effect is neutropenia; however, our patient developed transaminitis. The novelty of our case is the development of hepatotoxicity even after the introduction of another CDK 4/6 inhibitor, indicating at least some degree of class effect. In summary, CDK 4/6 inhibitors have significantly improved outcomes in hormone-positive metastatic breast cancers. However, a small percentage suffer from hepatic injury enough to warrant discontinuation of the drug, and we must continue to assess the risk versus benefit profile when offering them to our patients.

## Introduction

Cancer cells use various mechanisms to proliferate. One such mechanism involves evasion of immune system detection. This entails antigen alteration, cytokine alteration, and upregulation of proteins involved in T-cell suppression [1]. A focus of therapy within immune system evasion involves cyclin-dependent kinases (CDKs), which are a family of kinases involved in progression within the cell cycle. In breast cancer, the CDK 4/6 axis has been shown to be particularly hyperactive, and blockade of this axis has shown promising results in preventing tumor cell growth [2]. Previously, hormone-positive metastatic breast cancer was treated with endocrine therapy and chemotherapy. Now, with the introduction of CDK 4/6 inhibitors, the way tumor cells exploit the protection of the immune system is circumvented and has forever changed the landscape of therapy for metastatic breast cancer. Palbociclib is a CDK 4/6 inhibitor that has been shown to significantly improve progression-free survival rates when combined with letrozole in the PALOMA-2 trial [3]. Similarly, ribociclib and abemaciclib are two other CDK 4/6 inhibitors that have showed improved outcomes against placebo in the MONALEESA-2 trial and the MONARCH trial, respectively [3,4]. While CDK 4/6 inhibitors are generally well tolerated, there are notable adverse effects. Due to extensive hepatic metabolism through the CYP3A4A pathway, CDK 4/6 inhibitors have been shown to have a serious effect on liver enzymes [5-7]. For ribociclib, grade 3 hepatotoxicity was seen within an average of 85 days and did not improve to grade 2 until approximately 22 days post-discontinuation [6]. As a result, liver function test (LFT) monitoring is recommended [6]. Similarly, abemaciclib has been shown to cause grade 3 hepatotoxicity within 60 days and will usually take 14 days to resolve below grade 3; LFT monitoring is recommended [6]. Palbociclib is less likely to cause hepatotoxicity, but case reports have reported this adverse effect [8,9]. We present a case in which hepatotoxicity was seen after initiation of ribociclib, and subsequently with palbociclib, for hormone-positive HER2-negative metastatic breast cancer.

## Case presentation

**Figure 1:**
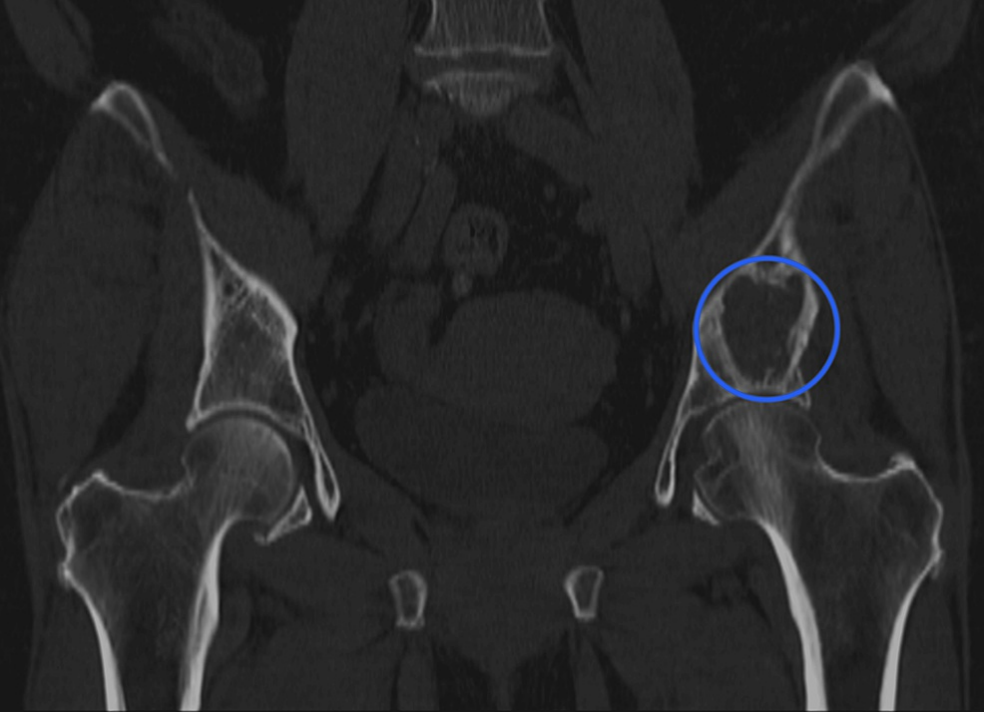
Coronal non-contrast computed tomography image of the pelvis demonstrating a lytic lesion (blue circle) within the left ilium acetabulum.

We present a case of a 67-year-old female with a past medical history of monoclonal B-cell lymphocytosis, and a resected dermatofibrosarcoma of the left medial thigh, who was diagnosed with stage 1C invasive ductal carcinoma 12 years ago. She did not receive adjuvant chemotherapy due to a low-risk oncotype DX score and was thus treated with lumpectomy and radiation. She subsequently took tamoxifen for five years. She then began experiencing left hip pain a couple of years ago, was diagnosed with bursitis, and received physical therapy and steroid injections. During further evaluation of her hip, computed tomography (CT) imaging demonstrated a left ilium lytic lesion 6.0 x 2.5 x 4.5 cm extending into the left acetabulum (Figure 1), as well as an indeterminate sclerotic lesion of the left sacral ala consistent with a bone island.

**Figure 2:**
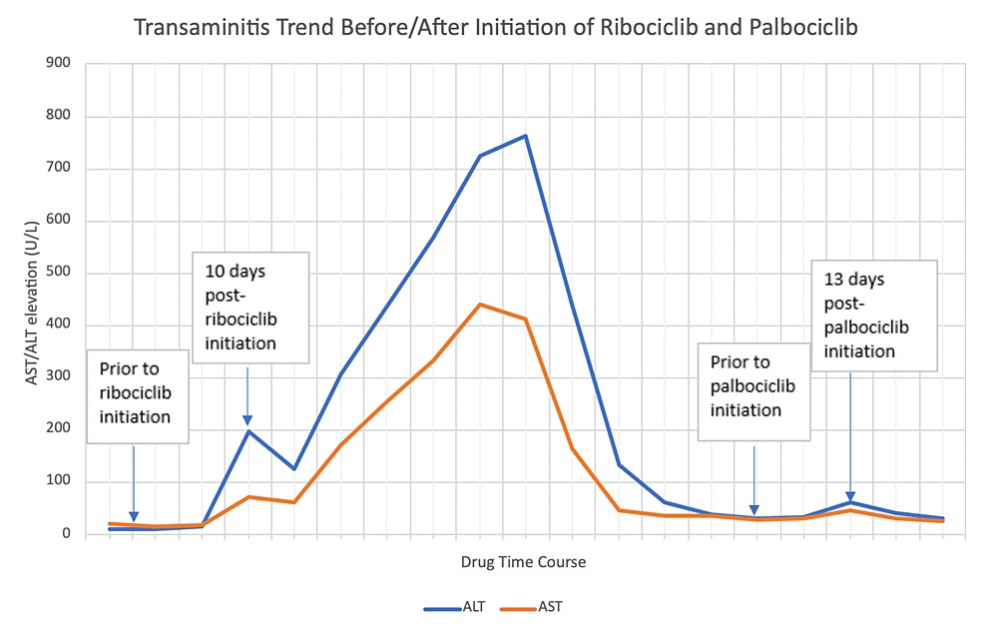
Chart showing trend of ALT and AST levels in relation to initiation of ribociclib and palbociclib. ALT, alanine transaminase; AST, aspartate transaminase

Left acetabular core needle biopsy revealed metastatic adenocarcinoma with features consistent with breast primary, estrogen receptor 100%, progesterone receptor 60%, and HER2 negative. She was started on letrozole and ribociclib. However, the patient developed elevated transaminases within 10 days of initiation of ribociclib with an alanine transaminase (ALT) of 197 U/L (normal range: 7-55 U/L) and aspartate transaminase (AST) of 71 U/L (normal range: 8-48 U/L), which later peaked with ALT of 762 U/L and AST of 411 U/L before starting to improve (Figure 2).

**Figure 3:**
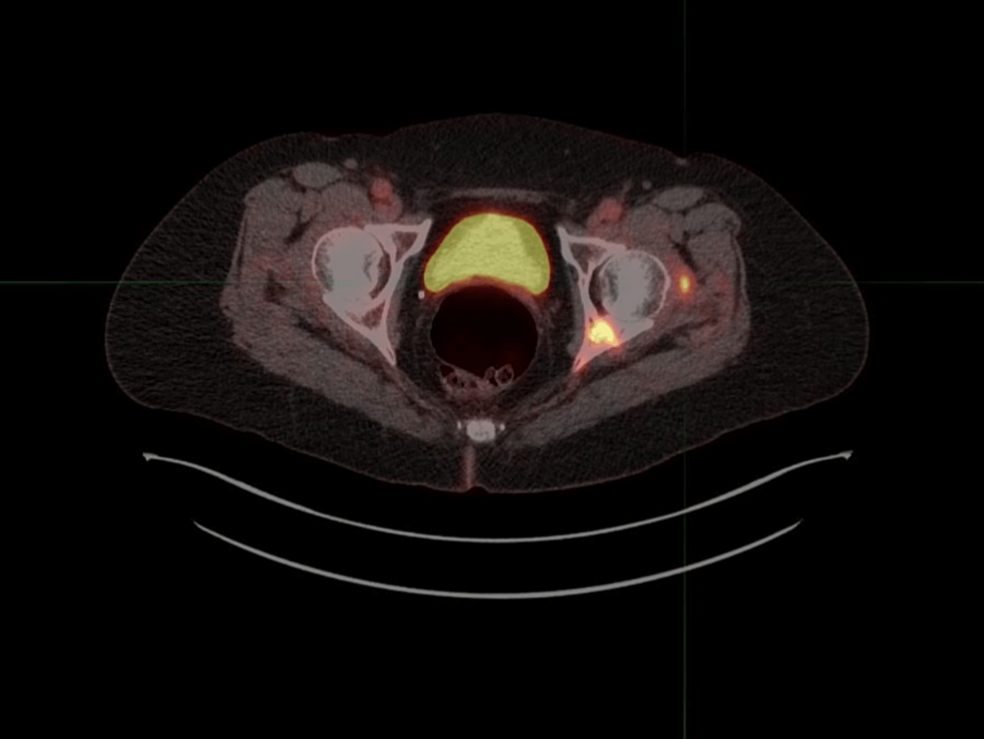
Positron emission tomography scan showing fluorodeoxyglucose avid progression of disease predominantly within the left posterior acetabular lesion and the soft tissue lesion adjacent to the left hip joint.

Ribociclib was held, but it took two months for the LFT to normalize. Total bilirubin and alkaline phosphatase were within normal limits. The patient was evaluated by the hepatology service, and she was tested for viral hepatitis, which was negative. She did not have signs or symptoms suggestive of hepatic decompensation. Other LFT including bilirubin and alkaline phosphatase were within normal limits. She had no prior history of alcohol abuse or autoimmune disease. She scored a 5 on the Naranjo Adverse Drug Reaction Probability Scale, indicating probable drug-induced liver injury, and thus her hepatology evaluation suspected her elevated transaminases to be drug-induced. Confirmatory liver biopsy was considered; however, given high suspicion for drug-induced liver injury, it was deferred. Ribociclib was thus discontinued, and she was started on palbociclib; however, within two weeks, she had again elevated ALT (62 U/L) and AST (45 U/L), needing discontinuation of palbociclib, and only letrozole was continued. Her LFT normalized after two weeks. She remained stable on letrozole for a year. Subsequent positron-emission tomography/CT imaging showed disease progression (Figure 3), and she was started on fulvestrant as a second-line treatment.

We contemplated adding abemaciclib at this time, but the patient refused to consider it, and she has had stable disease for more than a year on fulvestrant alone. 

## Discussion

CDK 4/6 inhibitors are one of the relatively newer therapies being used to treat metastatic breast cancer and are generally well tolerated. The most common side effect of CDK 4/6 inhibitors is neutropenia: 80% with palbociclib, 75% with ribociclib, and 41% with abemaciclib [6]. However, our patient was unique in that she developed elevated transaminases. The PALOMA-2 trial reported elevated transaminases as an adverse effect of palbociclib 43%-52% of the time, with grade 3-4 toxicity (defined as >5-20 upper limit of ALT/AST) occurring only 2%-3% of the time [6,10]. With ribociclib, elevated transaminases were seen in the MONALEESA trial 44%-46% of the time, yet grade 3-4 toxicity occurred 7%-10% of the time [6]. With abemaciclib, elevated transaminases occurred 37%-48% of the time, with grade 3-4 toxicity occurring 4%-6 % of the time [6]. Ribociclib is notorious for causing elevated transaminases, and thus LFT monitoring is encouraged; when elevated transaminases do occur, dose reduction or discontinuation is recommended [6]. Our patient developed toxicity quickly and needed a long period of time to recover, far longer than average [6]. To our knowledge, only a few cases have been reported with similar results. Topcu et al. report a case in which a patient developed fulminant hepatitis after ribociclib initiation, confirmed by liver biopsy [11]. However, this patient had elevation in bilirubin levels in addition to elevated transaminases, while our patient had normal bilirubin levels. Moreover, our patient developed hepatotoxicity again even after LFT normalization and introduction of another CDK 4/6 inhibitor, palbociclib. Interestingly, a case has been reported in which a patient developed grade 3 hepatotoxicity after ribociclib; however, the patient was successfully rechallenged with palbociclib using dose escalation without the resurgence of any hepatic injury [12]. Another case had a similar trajectory where hepatic injury was seen within three cycles of ribociclib initiation, treated with steroids with normalization of aspartate transaminase/alanine transaminase, and then successfully started on palbociclib with no subsequent evidence of hepatic injury [13]. Both of these cases illustrate successful treatment with another CDK 4/6 inhibitor, while our patient did not, indicating at least some degree of class effect.

## Conclusions

CDK 4/6 inhibitors have been proven to improve outcomes in hormone-positive metastatic breast cancers. While many patients are treated with these agents without experiencing adverse effects, some do, in fact, suffer from hepatic injury enough to warrant discontinuation of the drug. Furthermore, some experience fulminant hepatitis and require additional invasive testing including biopsy. Thus it is imperative that we continue to report adverse effects from these agents so that we can better assess the risk versus benefit profile when offering them to our patients.
